# Evaluation of osteogenic properties of a novel injectable bone-repair material containing strontium *in vitro* and *in vivo*

**DOI:** 10.3389/fbioe.2024.1390337

**Published:** 2024-04-19

**Authors:** Lishuang Liu, Sha Hou, Guangya Xu, Jingjing Gao, Junyu Mu, Min Gao, Jianrong He, Xiaoyu Su, Zheng Yang, Yi Liu, Tengzhuo Chen, Zhihong Dong, Lijia Cheng, Zheng Shi

**Affiliations:** Clinical Medical College, Affiliated Hospital, School of Basic Medical Sciences of Chengdu University, Chengdu, China

**Keywords:** hydroxyapatite, strontium, chitosan, bone regeneration, osteoporosis

## Abstract

**Objective:** This study aims to develop and evaluate the biocompatibility and osteogenic potential of a novel injectable strontium-doped hydroxyapatite bone-repair material.

**Methods:** The properties of strontium-doped hydroxyapatite/chitosan (Sr-HA/CS), hydroxyapatite/chitosan (HA/CS) and calcium phosphate/chitosan (CAP/CS) were assessed following their preparation via physical cross-linking and a one-step simplified method. Petri dishes containing *Escherichia coli* and *Staphylococcus epidermidis* were inoculated with the material for *in vitro* investigations. The material was also co-cultured with stem cells derived from human exfoliated deciduous teeth (SHEDs), to assess the morphology and proliferation capability of the SHEDs, Calcein-AM staining and the Cell Counting Kit-8 assay were employed. Osteogenic differentiation of SHEDs was determined using alkaline phosphatase (ALP) staining and Alizarin Red staining. For *in vivo* studies, Sr-HA/CS was implanted into the muscle pouch of mice and in a rat model of ovariectomy-induced femoral defects. Hematoxylin-eosin (HE) staining was performed to determine the extent of bone formation and defect healing. The formation of new bone was determined using Masson’s trichrome staining. The osteogenic mechanism of the material was investigated using Tartrate-resistant acid phosphatase (TRAP) staining and immunohistochemical studies.

**Results:** X-ray diffraction (XRD) and energy-dispersive spectroscopy (EDS) showed that strontium was successfully doped into HA. The Sr-HA/CS material can be uniformly squeezed using a syringe with a 13% swelling rate. Sr-HA/CS had a significant antibacterial effect against both *E. coli* and *S. epidermidis* (*p* < 0.05), with a stronger effect observed against *E. coli*. The Sr-HA/CS significantly improved cell proliferation and cell viability *in vitro* studies (*p* < 0.05). Compared to CAP/CS and CS, Sr-HA/CS generated a substantially greater new bone area during osteoinduction experiments (*p* < 0.05, *p* < 0.001). The Sr-HA/CS material demonstrated a significantly higher rate of bone repair in the bone defeat studies compared to the CAP/CS and CS materials (*p* < 0.01). The OCN-positive area and TRAP-positive cells in Sr-HA/CS were greater than those in control groups (*p* < 0.05).

**Conclusion:** A novel injectable strontium-doped HA bone-repair material with good antibacterial properties, biocompatibility, and osteoinductivity was successfully prepared.

## 1 Introduction

Osteoporosis, a prevalent global health concern, affects millions of individuals worldwide and poses a significant medical challenge. This systemic skeletal disorder is characterized by reduced bone mass, compromised microstructural integrity leading to increased bone fragility, and heightened susceptibility to fractures. Consequently, the management of osteoporosis often necessitates the utilization of bone repair materials for the treatment of pathological fractures across diverse anatomical sites. Autogenous bone transplantation is considered the most effective method for the treatment of large-scale bone defects resulting from trauma, infection, tumor, or ischemic osteonecrosis (Silva Filho et al., 2013; Ahn et al., 2014). However, the clinical application of autogenous bone is greatly limited due to limited sources and the risk of infections and immune rejection (Myeroff and Archdeacon, 2011). Therefore, there is an urgent need for bone tissue–engineering materials in clinical settings as an alternative to implant materials. Traditional biomaterials such as hydroxyapatite (HA) and calcium phosphate (CAP) have been extensively investigated because of their superior bone conductivity, biocompatibility, and biodegradability (Gao et al., 2014). However, these materials have certain drawbacks, including limited biological activity and poor mechanical properties that prevent them from adapting to the shape of the defect (Rezwan et al., 2006; Mercado-Pagán et al., 2015). Furthermore, the majority of calcium phosphate-containing materials lack antibacterial activity. This limitation restricts their use in bone repair (Campoccia et al., 2006; Yuan et al., 2014).

Fortunately, the discovery of strontium (Sr) has invoked renewed interest in conventional calcium phosphate–containing materials. Sr is a trace element found in humans’ body in amounts ranging from 0.008% to 0.01%, which is widely distributed in human hard tissues, such as bones and teeth. Due to the similar ionic radius of strontium ions (Sr^2+^) and calcium ions (Ca^2+^) (0.12 nm vs. 0.099 nm), Sr^2+^ can frequently displace calcium ions Ca^2+^ in HA (Boanini et al., 2010). Geng et al.demonstrated that Sr could completely replace Ca and that the addition of Sr increased the lattice parameters of HA and enhanced the biological activity (Geng et al., 2016; Geng et al., 2018). In addition to being an essential constituent of bone tissue, Sr regulates the physiological environment of cells and promotes bone healing (Glenske et al., 2018). It is recognized for its distinctive dual mechanism of action, which simultaneously promotes bone formation and hinders bone resorption (Bonnelye et al., 2008). Clinically, Sr ranelate has been utilized for the treatment of osteoporosis in postmenopausal women (Meunier et al., 2004; Meunier et al., 2009). However, it may increase the incidence of side effects such as heart disease and thromboembolism, leading to its restriction by the European Medicines Agency in 2014 (Diepenhorst et al., 2018). Studies have indicated that Sr can stimulate calcium-sensitive receptors, MAPK ERK1/2, NFATc/Maf, and the Wnt pathway, which in turn enhances the process of osteoblast differentiation (Chattopadhyay et al., 2007; Saidak et al., 2012). Moreover, it can reduce osteoclast differentiation by inhibiting the NF-κB pathway (Caudrillier et al., 2010). Therefore, considering the effects of Sr on the physiological process of bone remodeling, a viable safe approach to enhance osseointegration could involve the combination of Sr with implants to stimulate bone differentiation properties (Li et al., 2010; Zhang et al., 2016; Wang et al., 2019). However, limited studies have been reported on Sr-doped compounds or related materials for bone regeneration.

In addition, it is necessary for the implanted biomaterials to have excellent biocompatibility and antibacterial activity, because the implanted biomaterials may trigger a dependent inflammatory response followed by infection, thereby leading to surgical failure. Fortunately, in many studies, chitosan (CS) has gradually been discovered to have excellent performance. CS is a naturally occurring polysaccharide with exceptional biocompatibility, biodegradability, and antibacterial properties, finds extensive application in bone tissue engineering (Madihally and Matthew, 1999; Di Martino et al., 2005; Venkatesan and Kim, 2010; Croisier and Jérôme, 2013). CS binds and combines with negatively charged substances on the bacterial surface, forming an impermeable layer that obstructs the transport of important solutes, due to the presence of multiple positively charged amine groups in its molecular structure (Khattak et al., 2019). Another method involves the ability of low-molecular-weight CS to enter cells, alter the structure of DNA, and hinder the production of RNA and proteins in bacteria (Li et al., 2022).

After the incorporation of Sr into HA, Sr^2+^ can replace the Ca^2+^ in HA and become Sr/HA. Moreover, the difference in ionic radius and properties between Sr^2+^ and Ca^2+^ will distort the crystal lattice of the original HA, thus changing the crystal structure and biodegradability of HA, so that it can better match and integrate with natural bone. CS hydrogels can enhance the biocompatibility and antibacterial properties of the materials, and can be used as scaffolds. The addition of Sr/HA microspheres not only enhanced the mechanical properties of hydrogel, but also effectively promoted the healing of bone defects. A simplified one-step approach was used in this study to prepare Sr-doped HA (Sr-HA) to enhance the osteogenic capabilities of the material. A composite CS/medical polyvinyl alcohol gel was prepared using a physical cross-linking approach to make it suitable for injection. Finally, the modified Sr-HA was incorporated into the CS gel to develop the novel Sr-HA/CS. The material characterization experiments, *in vitro* antibacterial tests, cellular experiments, and *in vivo* experiments were used to determine the characteristics of the synthesized materials and assess their clinical viability for the treatment of osteoporotic bone defect. It is anticipated that this composite material can enhance the potential for bone regeneration applications.

## 2 Materials and methods

### 2.1 Fabrication of the Sr-HA/CS

**FIGURE 1:**
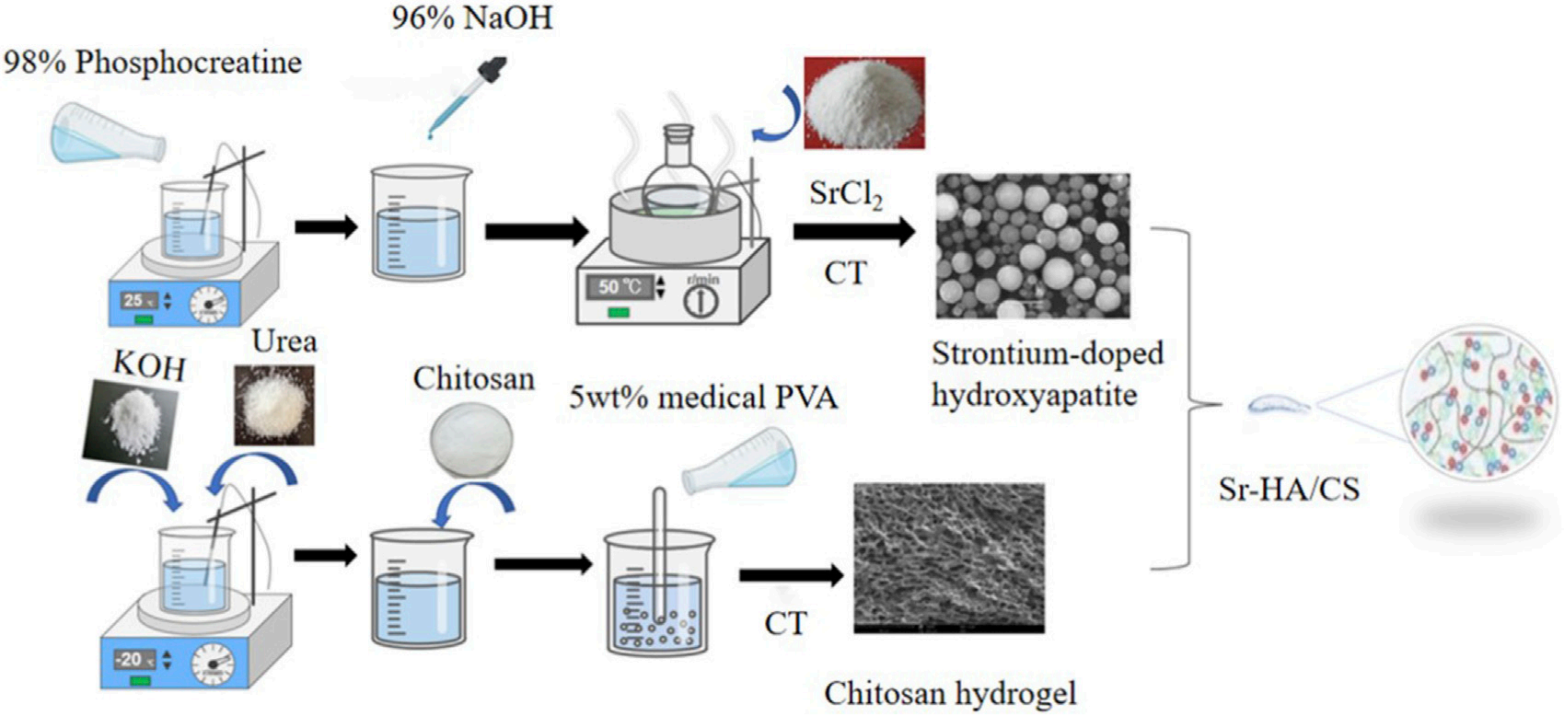
One-step simplified method and physical cross-linking were used to prepare Sr-HA/CS. (1) 98% phosphocreatine was mixed with 96% NaOH and heated followed by the addition of SrCl_2_ to obtain strontium-doped hydroxyapatite (Sr-HA); (2) KOH, urea, chitosan, and 5wt% medical PVA were added separately and the gel was prepared after thawing and freezing cycle; (3) Finally, a specific proportion of the two substances were mixed to produce Sr-HA/CS.

A 50 mL solution of phosphocreatine (98%, Hefei Bomei Biotechnology, Anhui, China) was added dropwise into a 50 mL solution of calcium chloride (99%) to prepare a mixed solution. The resulting solution was then stirred using a magnetic stirrer (WH220-HT, WIGGENS, Germany) for 30 min at 1,200 r/min. The pH of the solution was adjusted to 10 using a NaOH solution. After that, 0.525 g of SrCl_2_ (99% purity, Macklin, Shanghai, China) was added to the solution, which was then subjected to heating in an oil bath at 120°C for 30 min. The solution was subsequently cooled to room temperature, allowed to precipitate, filtered using a filter paper, washed twice with phosphate-buffered saline (PBS), and freeze-dried in a vacuum freeze drier (FDU-2100, EYELA, Japan) to obtain Sr-HA microspheres. Simultaneously, CS hydrogel was made using the following procedure: 16 g KOH and 8 g urea were accurately measured in a balance and added to 71 mL distilled water. The solution was decanted into a 150-mL flask, stirred slowly with a glass rod, and cooled to 20°C in a refrigerator. Then, 5 g of CS (degree of deacetylation ≥95%, Macklin, Shanghai, China) was introduced into the solution and stirred at 1,200 r/min for 1 h using a magnetic stirring to produce a transparent CS solution. Following this, 100 g of medical polyvinyl alcohol (5%, Evoh, Japan) was added to the CS solution, which was stirred at 25°C for 30 min at 1,200 r/min with a magnetic heating agitator. The CS/medical polyvinyl alcohol solution was heated in a water bath at 50°C for 1 h. The solution was frozen at −20°C for 12 h and thawed at room temperature (25°C). The freeze-thaw cycle was repeated three times. The resulting solution was dialyzed in distilled water for 5 days and then filtered to remove the residue to get the CS hydrogel. Finally, the CS gel and HA microspheres were mixed in a specific solid-liquid ratio (0.05–0.2 g/mL) and stirred for 1 h with a magnetic stirrer at 1,200 r/min to produce a homogenous, milky composite hydrogel of the Sr-HA/CS composite (Figure 1). Furthermore, control trials were conducted using HA/CS and CAP/CS materials without Sr. All materials were exposed to 250 nm UV light (Chuangu Lighting Technology Co., Ltd.) for an hour before implantation.

### 2.2 Characterization of materials

#### 2.2.1 Surface and composition analyses of Sr-HA and HA

To evaluate surface morphology of Sr-HA and HA, the sample was sprayed with gold in a vacuum for 10 min and then examined using scanning electron microscopy (SEM, Regulus8100, Hitachi, Japan). After gold plating, the elements of the material were identified using energy dispersive spectroscopy (EDS, Regulus8100, Hitachi, Japan). The sample was analyzed using X-ray diffractometry (XRD, D8 ADVANCE, Bruker, Germany).

#### 2.2.2 Swelling of Sr-HA/CS and HA/CS

To evaluate the swelling rate (SR), the Sr-HA/CS and HA/CS hydrogel was dried at 60°C in a constant temperature vacuum dryer (DZF6020, China). A specific amount of the dried hydrogel was weighed (W1). Then, the hydrogel was soaked in PBS for 12 h. The liquid on the surface of the sample was removed and the sample was measured using an electronic balance (W2). The formula used to calculate SR is as follows: SR = (W2–W1)/W1.

#### 2.2.3 Injectability of Sr-HA/CS

The hydrogel was loaded in a 5-mL syringe and squeezed into a Petri dish containing PBS. The criteria for judgment were the ability of the gel to be squeezed out uniformly and the retention of its shape in solution.

### 2.3 Antibacterial effect of Sr-HA/CS hydrogel

The bacteriostatic effect of the Sr-HA/CS hydrogel was determined. First, 0.1 mL of *E. coli* (*Escherichia coli*) and *S. epidermidis* (*Staphylococcus epidermidis*) at a density of 2 × 10^6^ cells/mL was uniformly spread onto agar plates using a sterile swab. Four holes were punched in the agar plates using a pipette tip and the plugs in the medium were removed from the holes using a sterile needle. The plates were sealed to prevent contamination and incubated at a temperature of 37°C and then examined 18 h later. The diameters of the bacteriostatic circles were quantitatively measured with a Vernier caliper and the effects of bacteriostasis and colony growth were observed and recorded.

### 2.4 *In vitro* studies

#### 2.4.1 Cell culture

Following the sterilization of the two material groups (Sr-HA/CS and HA/CS), 2 mg/mL rat tail glue (Solarbio, China) was introduced and distributed across a 24-well plate (Nest, America). The plate was then subjected to ultraviolet gel. Next, 5 × 10^4^ stem cells derived from human exfoliated deciduous teeth (SHEDs) were placed on the Sr-HA/CS and HA/CS bioceramics in 24-well plates, and the culture medium was replaced after 24 h. The Dulbecco’s Modified Eagle Medium (high glucose) was supplemented with 1% penicillin-streptomycin and 10% standard fetal bovine serum.

#### 2.4.2 Cell proliferation

Cell proliferation was assessed using the Cell Counting Kit-8 (CCK-8, Biyuntian, China). Briefly, 2 × 10^4^ SHEDs were inoculated on samples in 48-well plates. The samples were then cultured for 1, 4, and 7 days, respectively, and incubated in CCK-8 working solution for 2 h. The culture medium was replaced every 2 days. Then, 100 μL of the sample solution was transferred to a 96-well plate and the absorbance was measured at 450 nm. The relative growth rate (RGR) was calculated using the following equation: RGR (%) = OD_test_/OD_control_ × 100%.

In morphological studies, cells were treated for 15–30 min with a Calcein-AM fluorescent dye kit (HR0444, Baiaolaibo, China) after 7 days of cell culture. The Calcein-AM staining solution was removed and the cells were washed twice with a serum-free medium. Following the preparation of Hoechst working solution (C1017, Biyuntian, China) at a concentration of 20 μg/mL, the cells were further incubated for 10–20 min. The cells were then washed twice with PBS before being examined under inverted fluorescence microscopy (IX73, Olympus, Japan). RGR was calculated using OD_control_ represents the absorption of the sample at 12 h.

#### 2.4.3 Alkaline phosphatase (ALP) activity

The cells were inoculated into each well at a density of 3 × 10^4^ cells. Osteoinduction commenced as the cells cultivated on the tissue culture plate achieved 80% confluence, the materials were added in the cell to induce cell osteogenic differentiation. The samples were transferred to a new well plate and washed with PBS after 7 and 14 days. The cells on the sample were lysed in 200 μL of 0.1% polyethylene glycol octyl phenyl ether (Tritonx-100) buffer for 30 min, and the lysate was collected in a 1.5-mL microcentrifuge tube. The ALP activity in the supernatant was measured using an ALP assay kit (YX-W-B002, Ainobestbio, China). The total protein content was evaluated using a bicinchoninic acid assay kit, following the instructions provided by the manufacturer. The ALP activity was determined and calibrated using the total protein content (U/g).

#### 2.4.4 Extracellular matrix mineralization

After 21 days of osteogenic induction, Alizarin Red staining was performed to evaluate the surface mineralization level of samples. Briefly, the samples were fixed in 4% paraformaldehyde for 30 min, washed with PBS, and then stained with Alizarin Red staining solution (Jiayuan Biotechnology, Guangzhou, China) for 15 min. The samples were then thoroughly rinsed with distilled water and images were captured with a stereoscopic microscope (Leica, Germany). After 15 min of elution in 1 mL of 10% cetylpyridinium chloride (Sinopharm Chemical Reagent, China), the mineralized body was transferred to a 96-well plate at 37°C and its absorbance at 562 nm was measured using semi-quantitative analysis. In general, materials with good biocompatibility exhibited more mineralized nodules.

### 2.5 *In vivo* studies

#### 2.5.1 Bone induction surgery in mice

All animal experimental protocols were approved by the Animal Ethics Committee of Chengdu University. Surgery was conducted on animals after 1 week of adaptive housing. Twenty-four 8-week-old female ICR mice (Chengdu Dashuo Experimental Animal Co., Ltd., China) were randomly divided into the following four groups (*n* = 6 each): 0.01 mL of CS, 0.01 mL of Sr-HA/CS, 10 μg of calcium phosphate (CAP), and 0.01 mL of CAP/CS. Calcium phosphate-based materials are very similar to the inorganic components of human bone, both in terms of chemical composition and biological properties, and are therefore widely used in bone tissue engineering and clinical medicine. In our study, we chose to increase the CAP group for comparison, thus better demonstrating the advantages of our prepared materials. Before implantation, CAP, an amorphous powder, was moistened with sterile PBS and thereafter underwent natural drying and solidification. The ratio of Sr HA/CAP: CS hydrogel was 0.05 g: 10 mL. The mice were anesthetized using isoflurane gas (Shenzhen Reward Life Science and Technology Co., Ltd.). After complete anesthesia, the hair on both outer thighs of mice was clipped, the skin was sterilized with ethanol, and a 10-mm long incision was made in the skin. Subsequently, a longitudinal muscle pocket measuring approximately 8 mm in length was promptly prepared along the skin incision. Finally, the four kinds of materials were implanted, and the muscles and skin were sutured sequentially. Normal postoperative feeding was performed and penicillin was injected for 3 consecutive days to prevent postoperative infection. Following the CO_2_-inhalation-induced execution of mice at 8 and 10 weeks, the tissues were fixed for 72 h in 4% paraformaldehyde.

#### 2.5.2 Bone-defect surgery in OVX rats

Thirty 3-month-old female Sprague Dawley rats weighing >200 g (Chengdu Dashuo Experimental Animal Co., Ltd., China) were bilaterally ovariectomized after 1 week of acclimatization. After 8 weeks, OVX rats were placed in a supine position on a heating pad to ensure their body temperature was maintained at 36°C–37°C after anesthesia and skin preparation. The hind limbs of OVX rats were shaved and disinfected, and an incision of approximately 1.5 cm in length was made in the skin of the distal femur to expose the muscle. After a blunt dissection of the muscle to expose the femoral condyles, a circular bone defect of about 3 mm in diameter was drilled perpendicular to the median axis with a medical DC drill at a low speed of 3,000 rpm. To prevent thermal necrosis of cells and tissues, 0.9% saline was continuously flushed during drilling. After drilling, bone fragments were removed from the cavity by rinsing with saline solution. The skin was then sutured using each of the four materials filled into the hole. To avoid infection, penicillin was administered intraperitoneally in three consecutive injections postoperatively. The execution method and time points were similar to those used for mice.

#### 2.5.3 Histological staining

Specimens were decalcified in 10% ethylenediaminetetraacetic acid for 3 weeks at room temperature while being subjected to agitation on a shaker. Specimens were dehydrated in a series of ethanol solutions with concentrations ranging from 75% to 100%, embedded in a paraffin-embedding machine (TKD-BMC, Hubei, China), and sliced into 5-µm-thick posterior sections using a microtome (RM2235, Leica, Germany). Following that, hematoxylin-eosin (HE) staining, Masson’s trichrome staining, saffron-solid green (S&G) staining, and tartrate-resistant acid phosphatase (TRAP) staining were performed separately according to the manufacturer’s instructions. The images were scanned using a NanoZoomer digital pathology scanner (NDP; Hamamatsu, Japan). Three slices were randomly selected based on HE staining, and new bone areas were automatically measured and calculated using Image-Pro Plus 6.0 (IPP). The formula used to calculate new bone area is as follows: New bone area (%) = new bone area/tissue area × 100%.

#### 2.5.4 Immunohistochemistry

The paraffin sections were deparaffinized, washed, and incubated in antigen repair solution before being treated with hydrogen peroxide. The prepared primary antibody [OCN (1:100, Servicebio)] was added dropwise and incubated overnight at 4°C. The secondary antibody, specifically horseradish peroxidase–labeled rabbit anti-goat IgG (1:200, Servicebio), was introduced and allowed to incubate for 50 min at room temperature. This was followed by the gradual addition of the DAB color development solution and hematoxylin. Positive staining was then quantified using IPP and represented as a percentage of positive area.

### 2.6 Statistical analysis

Data are expressed as mean ± standard deviation ( $\bar{x}$ ± s) and analyzed using SPSS 26.0. The differences between multiple groups were analyzed using a one-way analysis of variance, while the differences between two groups were analyzed using an independent samples *t*-test. A *p* < 0.05 was considered statistically significant (*p < 0.05, **p < 0.01, ***p < 0.001).

## 3 Results

### 3.1 Characterization of materials

#### 3.1.1 Surface morphology and composition analysis of Sr-HA/CS

**FIGURE 2:**
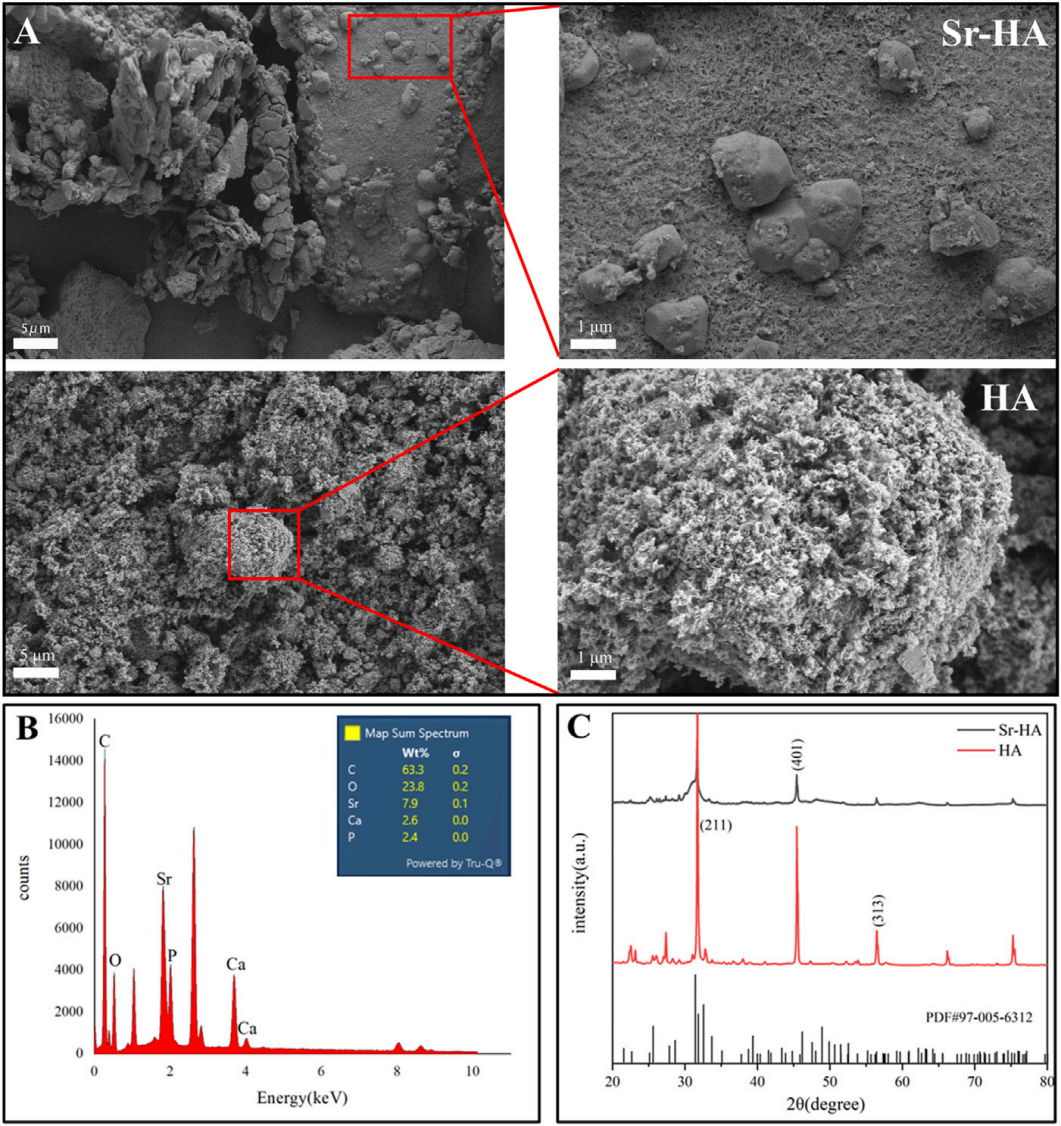
Surface morphology and composition analysis of Sr-HA and HA powder. **(A)** SEM images of Sr-HA and HA, **(B)** EDS analysis of Sr-HA, and **(C)** XRD pattern of Sr-HA and HA powder.

Figure 2 illustrates the morphology and EDS element mapping of Sr-HA powder. SEM analysis revealed the presence of large spheroidal structures on the surface of the powder, which differed from the typical HA structure (Figure 2A). Sr was successfully incorporated into the powder, as evidenced by EDS mapping (Figure 2B). The phase composition of the sample was analyzed using XRD and the most similar HA PDF card was searched using Jade 9.0. Compared to the standard card, in our analysis, the HA and Sr-HA generated showed similar characteristic peaks of HA, specifically at 211, 401, and 313. HA and Sr-HA powders had similar peaks at 31.6°, 45.4°, 56.4°, 66.2°, and 75.2°. However, the peak intensity of Sr-HA was lower, which may be related to the incorporation of Sr (Figure 2C).

#### 3.1.2 Injectability assay

**FIGURE 3:**
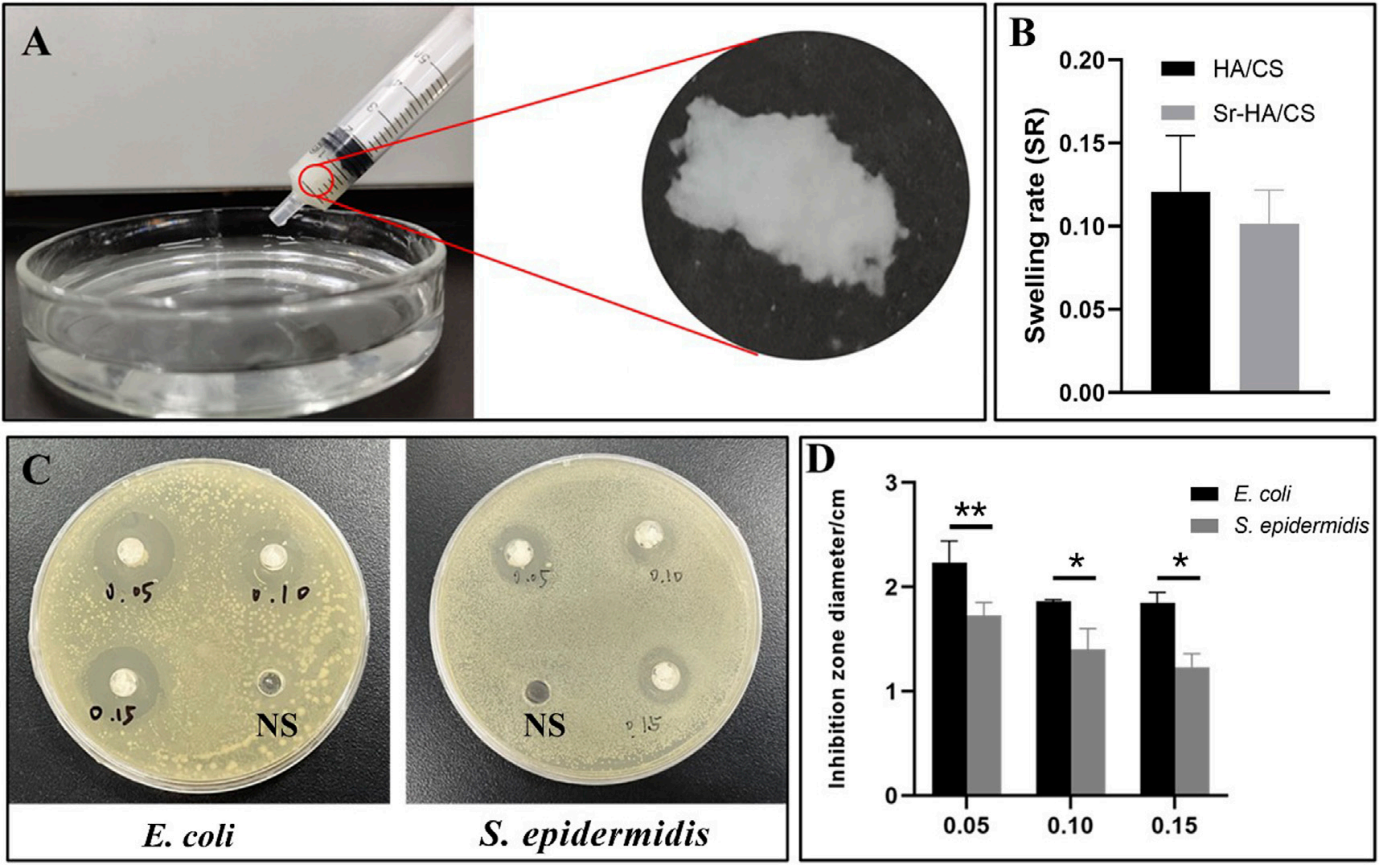
*The* tests on injectability, swelling and antibacterial properties of Sr-HA/CS gel. **(A)** Injectability of Sr-HA/CS. **(B)** Swelling experiments of Sr-HA/CS and HA/CS (*n* = 3). **(C)** The inhibitory impact of varying concentrations of Sr HA/CS against *E. coli* and *S. epidermidis*. **(D)** Inhibition zone of varying concentrations of Sr-HA/CS against *E. coli* and *S. epidermidis* (*n* = 3, *p < 0.05, **p < 0.01, upon comparison with HA/CS group).

The hydrogel was filled in a 5-mL syringe and squeezed evenly into a Petri dish containing PBS. The squeezed gel was observed in the Petri dish and it retained its original shape (Figure 3A).

#### 3.1.3 Swelling assay

The weight change of the hydrogel before and after 12 h was measured using an electronic balance, and the histogram of the swelling rate was calculated using the formula SR = (W2-W1)/W1. Through statistical analysis, we found that there was no significant difference in swelling rate between Sr-HA/CS and HA/CS (*p* > 0.05, Figure 3B), and the inclusion of Sr may not change the water absorption of the material.

### 3.2 Evaluation of the antibacterial activity of Sr-HA/CS

To assess the ability of the synthesized materials to inhibit bacterial growth, 0.3 mL of saline (NS) was injected into Petri dishes containing *E. coli* and *S. epidermidis* as control samples. Simultaneously, 0.3 mL of Sr-HA/CS in the ratios of 0.05 g/10 mL (0.05), 0.10 g/10 mL (0.10), and 0.15 g/10 mL (0.15) were added. After 18 h of incubation, a noticeable zone of inhibition was observed around the material, in contrast to the control NS (Figure 3C). The statistical analysis of the diameters of the zones of inhibition, measured using the Vernier calipers, demonstrated that the material exhibited greater efficacy against *E. coli* at all ratios, as depicted in Figure 3D. The concentration of Sr-HA powder had a negligible impact on the inhibition effect at ratios of 0.05 and 0.10 groups. However, between 0.05 and 0.15 groups, the former exhibited a more pronounced inhibition effect, suggesting that Sr does not appear to exert an inhibitory effect in this experiment.

### 3.3 The osteogenic capacity assay of Sr-HA/CS *in vitro*

#### 3.3.1 Cell proliferation

**FIGURE 4:**
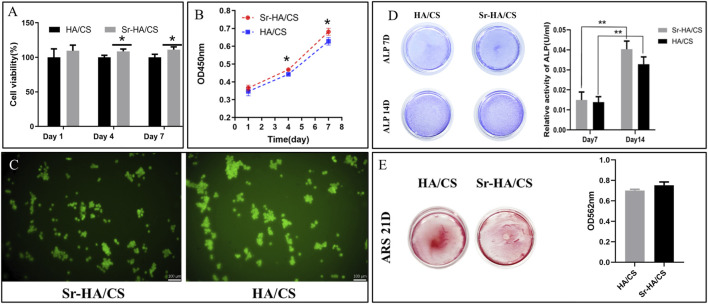
**(A)** Cell viability of Sr-HA/CS and HA/CS (*n* = 3) on days 1, 4, and 7. **(B)** CCK-8 assay revealing the proliferation capability of SHEDs of the two groups (*n* = 3). **(C)** Calcein-AM staining following 7 days and inverted fluorescence microscopy for the visualization of cell fluorescence. **(D)** ALP staining of SHEDs after 7 and 14 days of osteoinduction, and quantitative results of ALP staining (*n* = 3). **(E)** Alizarin Red staining of SHEDs after 21 days of osteoinduction, and quantitative results of Alizarin Red staining (*n* = 3). (ALP, alkaline phosphatase; *p < 0.05, **p < 0.01; compared with the HA/CS group).

The ideal bone-repair material should possess biocompatibility in addition to effective antimicrobial properties. Therefore, cell viability and proliferation of the materials cultured on days 1, 4, and 7 were assessed using the CCK-8 assay. The results of the CCK-8 assay indicated that there was no statistically significant difference in cell proliferation between the groups (*p* > 0.05) on day 1. However, the Sr-HA/CS group exhibited greater cell viability than the HA/CS group on days 4 and 7 (*p* < 0.05, Figure 4A). There was a noticeable upward trend in cell proliferation for both groups on days 1, 4, and 7. However, the Sr-HA/CS group exhibited a higher level of cell proliferation compared to the HA/CS group (*p* < 0.05, Figure 4B). Calcein-AM staining revealed that SHEDs could proliferated normally on the surface of the hydrogel (Figure 4C).

#### 3.3.2 ALP activity

ALP staining is indicative of the impact of early osteogenic differentiation on cells, while Alizarin Red staining can be used to assess the level of mineralization in late osteogenic cells. After 7 days of culture, ALP staining was darker in the Sr-HA/CS group than in the HA/CS group (Figure 4D). Additionally, the Sr-HA/CS group exhibited higher ALP activity than the HA/CS group. The results of ALP staining and activity on day 14 were in line with the pattern observed on day 7.

#### 3.3.3 Extracellular matrix mineralization

Following a 21-day incubation period, the mineralized bodies underwent treatment. The absorbance at 562 nm in the Sr-HA/CS group (Figure 4E) was higher, however, there was no significant difference between the two groups (*p* > 0.05).

### 3.4 Ectopic bone formation of Sr-HA/CS *in vivo*

#### 3.4.1 Histological analysis

**FIGURE 5:**
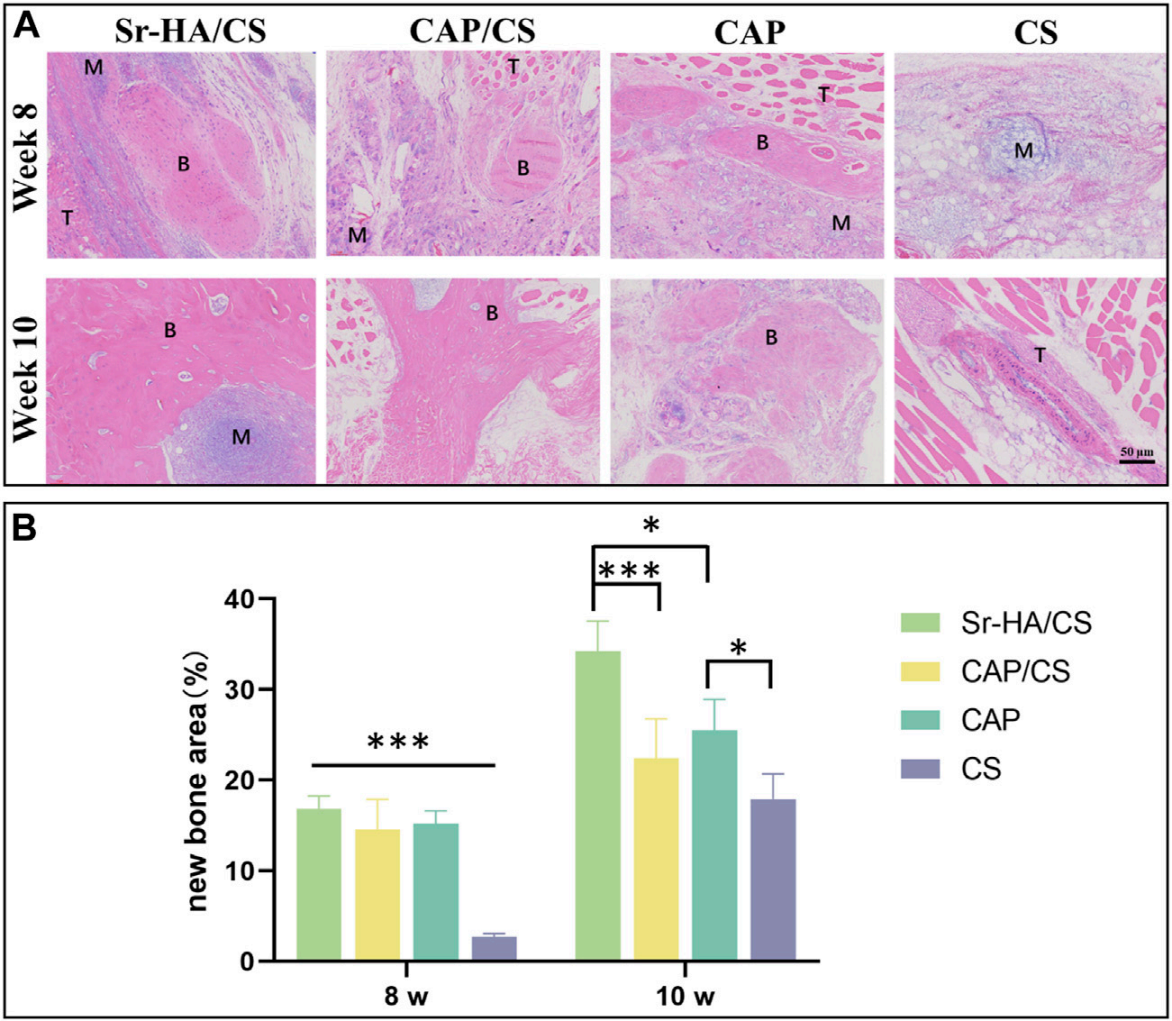
Histological analysis of osteoinduction in mice at weeks 8 and 10. **(A)** HE staining (M: material, T: tissue, B: bone; scale bar: 50 μm); **(B)** Quantitative analysis of the ratio of new bone area (*n* = 3, *p < 0.05, **p < 0.01, ***p < 0.001).

To investigate the osteoinductive ability of Sr-HA/CS, HE staining was performed on samples obtained from mice at 8 and 10 weeks after implantation. At 8 weeks, immature bone tissue was observed, whereas at 10 weeks, newly formed bone tissue was observed. Additionally, a part of the bone marrow tissue could be observed in the Sr-HA/CS group (Figure 5A). The new bone area of Sr-HA/CS, CAP/CS, and CAP all were greater than that of CS (*p* < 0.001), but did not differ significantly at 8 weeks (*p* > 0.05). At 10 weeks, the Sr-HA/CS group exhibited a greater new bone area compared to the other two groups (*p* < 0.05, *p* < 0.001). Furthermore, there was no significant difference in bone formation between the CAP/CS and CAP groups (*p* > 0.05, Figure 5B).

#### 3.4.2 Masson’s staining and TRAP analysis

**FIGURE 6:**
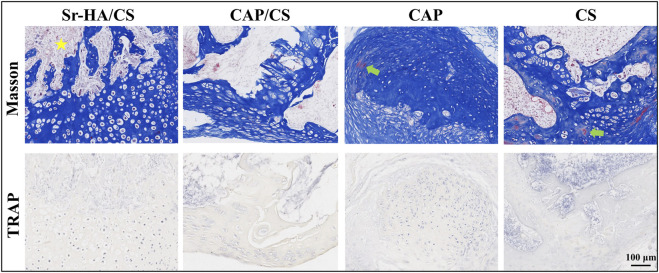
Masson’s staining and TRAP analysis in mice at week 10. Yellow pentagram: bone marrow tissue; green arrow: red staining, mature bone tissue; scale bar: 100 μm.

Serial sections of 10-week specimens were stained with Masson’s and TRAP to more clearly observe the development of new bone. At 10 weeks, the new bone was more mature and showed bone marrow tissue (Figure 6). Figure 6 illustrates that the absence of osteoclasts during TRAP staining suggests that the bone was in the early osteogenesis stage.

### 3.5 Repairing of bone defects with Sr-HA/CS in OVX rats

#### 3.5.1 Histological analysis

**FIGURE 7:**
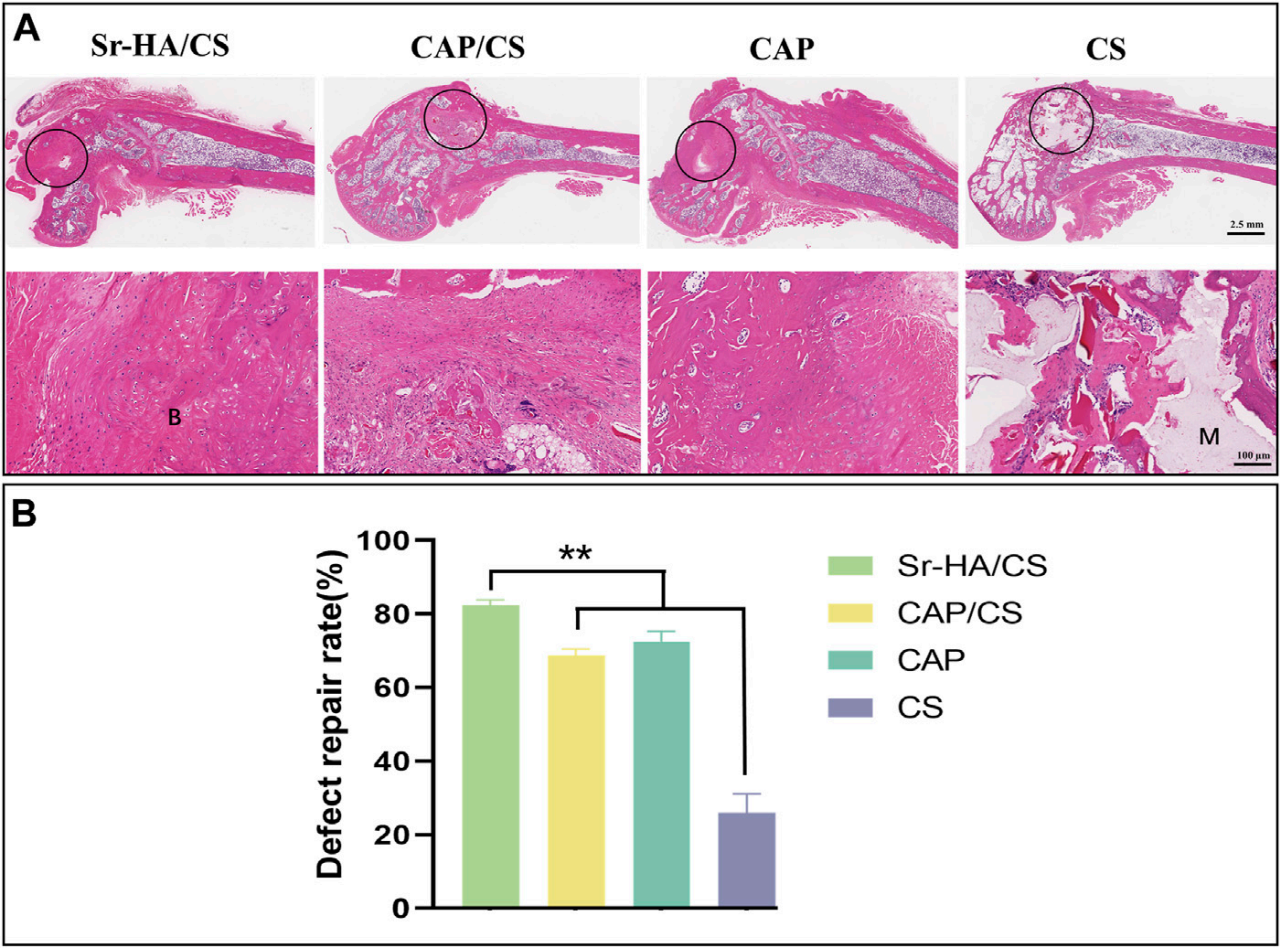
Histological analysis of bone defects in OVX rats at week 8. **(A)** HE staining (M: material, B: bone, black circle: bone-defect site; scale bar: 2.5 mm and 100 μm); **(B)** Quantitative analysis of defect repair rate (*n* = 3, **p < 0.01).

To assess the degree of osteoporosis and observe the healing process at the site of the bone defect, HE staining was applied. The Sr-HA/CS and CAP groups had superior healing as observed by the naked eye (Figure 7A), which was further confirmed by quantitative analysis (Figure 7B). Meanwhile, the trabeculae of the femoral epiphysis of CS were broken and sparsely arranged (Figure 7A). The remaining three groups displayed superior conditions. The sections exhibited significant growth in which a substantial amount of new bone tissue was generated, including bone marrow tissue. A large number of osteoblasts were seen, including in the CS (Figure 7A). The bone-repair rates of the four groups were as follows: 82.28% ± 1.43%, 69.11% ± 1.59%, 74.61% ± 4.91%, and 24.53% ± 5.11%. The repair rate of the Sr-HA/CS group showed a statistically significant increase compared to both the CAP/CS and CS groups (*p* < 0.01), but was comparable to that of the CAP group (*p* > 0.05).

#### 3.5.2 Masson’s staining and S&G staining

**FIGURE 8:**
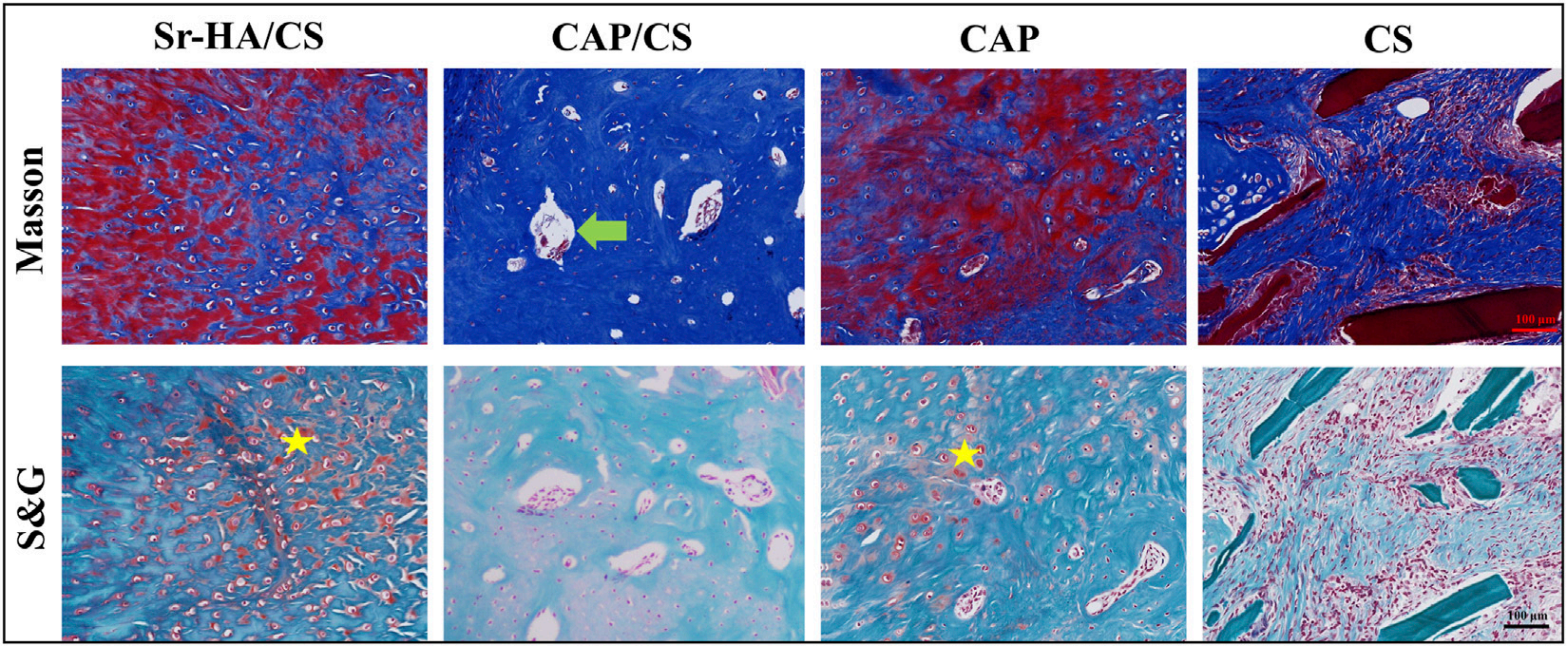
Masson’s and S&G staining of bone defects in OVX rats. Green arrow: bone marrow tissue; yellow pentagram: cartilage; scale bar: 100 μm.

Masson’s staining and S&G staining of serial sections of the specimens were used to visualize the new bone. Masson’s staining demonstrated the presence of distinct blue new bone, and the defects were replaced with lamellar bone and bone marrow. In comparison to the latter two groups, the Sr-HA/CS and CAP/CS groups had more area of new bone formation (Figure 8). The red portion (cartilage) observed in S&G staining suggested that the osteogenic process originated from the cartilage and progressed to the mature bone tissue; however, the defects were predominantly green (Figure 8), suggesting that the bone tissues were more mature at this time. In line with the findings of the Masson’s staining analysis, the Sr-HA/CS group showed the most pronounced osteogenesis, whereas the CS group did not demonstrate any obvious or substantial bone formation.

#### 3.5.3 Immunohistochemical and TRAP analyses

**FIGURE 9:**
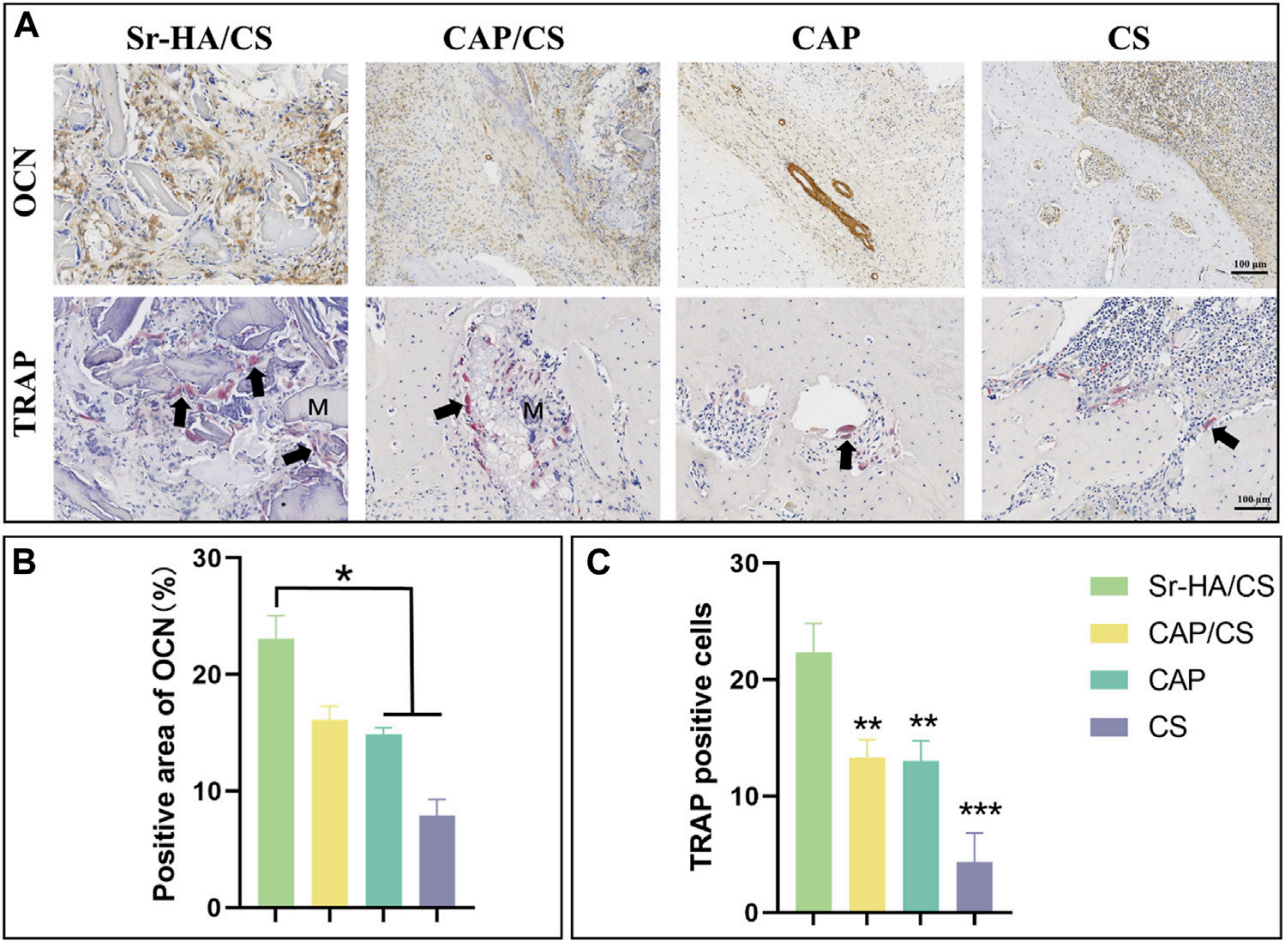
Immunohistochemical and TRAP analysis of bone defects in OVX rats. **(A)** OCN and TRAP staining (M: material, black arrow: osteoclast; scale bar: 100 μm); **(B)** Quantitative analysis of the positive area of OCN; **(C)** Quantitative analysis of TRAP-positive cells (*n* = 3, *p < 0.05, **p < 0.01, ***p < 0.001).

To elucidate the mechanism of osteogenesis, serial sections were stained with TRAP and OCN. During the 8-week, OCN-positive areas were observed both at the edges of the defect and in the central region of the newly formed bone (Figure 9A). The Sr-HA/CS group had the highest levels of positive staining, which were found to be considerably greater than those in the CAP and CS groups (*p* < 0.05, Figure 9B). Additionally, the TRAP staining of the multinucleate osteoblasts revealed that osteoblasts were considerably more abundant in the Sr-HA/CS group over the other three groups (Figure 9C), and these osteoblasts were found to be multinucleated (Figure 9A).

## 4 Discussion

Sr is not only a common component of bone, but also an essential trace element that plays a role in various physiological and biochemical processes in the human body (Pilmane et al., 2017). Recently, Sr has been extensively included in bioactive materials due to its exceptional bone-inducing capability and reduced adverse effects. For example, Cheng et al. (Cheng et al., 2023) prepared strontium ion-functionalized nano-hydroxyapatite/chitosan composite microspheres. The slow release of strontium ions from these microspheres facilitates the osteogenic differentiation of mesenchymal stem cells and the vascularization of endothelial cells, thereby promoting bone regeneration in defect areas. Geng et al. prepared titanium-based implants containing Sr. Through *in vitro* and *in vivo* experiments, they demonstrated that the incorporation of Sr promoted osteoblast adhesion and osteogenesis, inhibited osteoclastogenesis, increased bone formation and mineralization, and enhanced the bonding strength of bone implants (Geng et al., 2021; Geng et al., 2022). Studies have shown that Sr-doped biomaterials have superior safety and can induce bone formation and remodeling (Baron and Tsouderos, 2002; Marie et al., 2011; Neves et al., 2017).

The SEM analysis revealed that the Sr-HA powder showed a spheroid structure, while the XRD and EDS peaks confirmed the successful doping of Sr. Injectable biomaterials can provide better filling of defect shapes and therefore achieve better bone-to-material contact even for irregular defects (Kretlow et al., 2007). Furthermore, the XRD pattern demonstrated that the HA and Sr-HA prepared in this investigation exhibit comparable peaks associated with HA, which aligns with the findings reported by Hu et al. (Hu et al., 2020), and strontium doping does not significantly change the crystal structure of HA. Injectability experiments, which demonstrated that the material could be injected with a syringe, allowing the material to pass through the syringe into the bone-defect site in patients. In a clinical setting, injectable bone-repair materials may lessen the severity of surgically induced tissue fibrosis, reduce treatment expenses, and reduce patient discomfort (Cui et al., 2017). Furthermore, it would be beneficial to conduct additional research into the possibilities of improving mechanical properties through material ratio adjustments and minimizing solidification time *in vivo*.

The cell viability of the material is greater than 100% in all groups, suggesting exceptional biocompatibility of the material. Moreover, no significant cytotoxicity was observed, probably due to the excellent biocompatibility of CS and HA (Tetteh et al., 2014; Hao et al., 2020). Notably, at 4 and 7 days, cell viability was higher in the Sr-HA group, indicating a positive effect with Sr-HA and highlighting the beneficial effect of Sr ions (Pontremoli et al., 2022) in improving the biocompatibility of the material. Furthermore, extracellular ALP activity is an important parameter for determining osteogenic potential in both *in vivo* and *in vitro* (Prins et al., 2014). The ratio of inorganic phosphate (Pi) to inorganic pyrophosphate (Ppi) is crucial in the process of bone mineralization. ALP catalyzes the hydrolysis of Ppi into Pi. Additionally, Ppi inhibits the formation of HA. Therefore, ALP can be considered a reliable marker of bone mineralization (Vimalraj, 2020). At week 2, despite the lack of statistical significance of the results, ALP activity was higher in the Sr-HA group compared to the control group, which indicated Sr-HA had excellent osteogenic potential.

After the occurrence of a bone defect, the presence of a bacterial infection would inevitably impede the process of bone healing during treatment and implantation of biomaterials. Therefore, the implanted biomaterials may exhibit poor performance due to the inflammatory reactions at the site of the defect (Tenti et al., 2014; Neves et al., 2017). Therefore, biomaterials with excellent antibacterial efficacy and biocompatibility can effectively prevent inflammation resulting from bacterial infections. As previously mentioned in the introduction, numerous research has indicated that CS possesses antibacterial properties and can effectively inhibit the growth of several common pathogens. Germicidal efficacy against *E. coli* and *S. epidermidis* was significantly enhanced in this investigation with Sr-HA/CS, particularly against *E. coli*. Furthermore, the release of Sr^2+^ has the potential to impede bacterial activity, including processes such as growth and reproduction, cell wall formation, cell metabolism, and chromosomal replication (Enright et al., 2002; Brauer et al., 2013; Ranga et al., 2019). However, there is controversy regarding the antibacterial capabilities of Sr^2+^ (Sampath Kumar et al., 2015). The present study observed a decline in inhibition as the ratio of Sr-HA increased. This finding aligns with the research conducted by Kumar et al. (Sampath Kumar et al., 2015), which reported that calcium phosphate nanoparticles doped with Sr^2+^ exhibited limited antimicrobial activity against *S. epidermidis* and exhibited no effect against *E. coli*, even at a high concentration of 300 μg/μL. Moreover, Anastasiou et al. (Anastasiou et al., 2019) demonstrated that the antibacterial activity was reduced upon Sr^2+^ addition.

HA is an extremely biocompatible and essential mineral constituent of teeth and bone (Campana et al., 2014). The ability of HA to stimulate osteogenesis in muscle pouches of mice has been previously reported (He et al., 2022). Since calcium phosphate is also capable of inducing bone formation, it was utilized as a positive control while Sr-HA/CS was implanted into the muscle pouches of mice. HE staining revealed that the newborn bone of Sr-HA/CS was substantially different from that of the other three groups at 10 weeks, indicating that it exhibits a favorable osteoinductive property. In contrast, osteoclastogenesis was not detected using TRAP staining during a period of predominant new bone formation and when osteoclasts were not temporarily involved in bone remodeling.

Osteoporosis comprises a collection of bone metabolism disorders characterized by decreased bone mass and increased bone fragility (Wu et al., 2022), which may have a serious effect on the daily life of patients. OVX rats were used for bone-defect experiments. HE staining showed denser bone and more intact bone trabeculae in the Sr-HA/CS group. The Sr-HA/CS group had a significantly higher OCN positive area and TRAP cell count at week 8 compared to the other three groups, but the difference with respect to TRAP was more significant (*p* < 0.01). This could be attributed to the fact that Sr-HA/CS stimulates osteoclasts that promote bone formation during the phase of bone remodeling (Chen et al., 2022). OCN, a calcium-binding protein dependent on vitamin K, is a characteristic biomarker of osteoblasts (Liu et al., 2019). Therefore, it was anticipated that Sr-HA/CS might induce new bone formation via two stages: (1) Initial stage (0–8 weeks): It inhibits osteoclast formation and stimulates osteogenic differentiation of MSCs, thereby promoting OCN secretion; (2) In the late stage of implantation (≥8 weeks): By promoting bone remodeling, Sr-HA/CS facilitated the development of a substantial quantity of mature bone tissues enriched with active osteoclasts (Ranga et al., 2019). Additionally, various studies revealed the potential of Sr^2+^ to promote the proliferation and differentiation of mMSCs through the activation of membrane-bound calcium-sensitive receptors and Wnt/β-linker protein signaling pathways (Yang et al., 2011; Bose et al., 2013). Activation of the Wnt/β-linker protein pathway by Sr^2+^ ions liberated from Sr-HA/CS could potentially account for the enhanced secretion of proteins associated with osteogenesis. Furthermore, an *in vitro* study conducted by Lee et al. (Lee et al., 2021) showed that the incorporation of Sr^2+^ reduced the immune response to the material, thereby promoting bone regeneration *in vitro*. However, further research is required to comprehensively explain the mechanism through which Sr promotes osteogenic differentiation.

## 5 Conclusion

The current study reported the synthesis of novel injectable Sr-HA/CS with remarkable antibacterial properties against *E. coli* and *S. epidermidis*. *In vitro* studies including cell proliferation, CCK-8 assay, ALP activity, and extracellular matrix mineralization experiments demonstrated that Sr-HA/CS exhibits favorable characteristics for cell proliferation. Moreover, it was found to be non-toxic with the capability to promote osteogenesis. *In vivo* experiments provided evidence regarding the remarkable osteoinductive characteristics exhibited by Sr-HA/CS, as well as its potential in the treatment of osteoporosis and bone defects. Overall, the current findings revealed the potential of Sr-HA/CS to be used as a promising implant to repair bone defects at various sites through minimally invasive surgery.

## Data Availability

The raw data supporting the conclusion of this article will be made available by the authors, without undue reservation.
